# MCM-GINS and MCM-MCM interactions *in vivo *visualised by bimolecular fluorescence complementation in fission yeast

**DOI:** 10.1186/1471-2121-10-12

**Published:** 2009-02-19

**Authors:** Gökhan Akman, Stuart A MacNeill

**Affiliations:** 1 Department of Biology, University of Copenhagen, Copenhagen Biocenter, Ole Maaløes Vej 5, 2200 Copenhagen, Denmark; 2 Centre for Biomolecular Sciences, University of St Andrews, North Haugh, St Andrews, Fife KY16 9ST, UK

## Abstract

**Background:**

Each of the three individual components of the CMG complex (Cdc45, MCM and GINS) is essential for chromosomal DNA replication in eukaryotic cells, both for the initiation of replication at origins and also for normal replication fork progression. The MCM complex is a DNA helicase that most likely functions as the catalytic core of the replicative helicase, unwinding the parental duplex DNA ahead of the moving replication fork, whereas Cdc45 and the GINS complex are believed to act as accessory factors for MCM.

**Results:**

To investigate interactions between components of the CMG complex, we have used bimolecular fluorescence complementation (BiFC) in the fission yeast *Schizosaccharomyces pombe *for the first time, to analyse protein-protein interactions between GINS and MCM subunits expressed from their native chromosomal loci. We demonstrate interactions between GINS and MCM in the nuclei of exponentially-growing fission yeast cells and on chromatin in binucleate S-phase cells. In addition we present evidence of MCM-MCM interactions in diploid fission yeast cells. As with GINS-MCM interactions, MCM-MCM interactions also occur on chromatin in S-phase cells.

**Conclusion:**

Bimolecular fluorescence complementation can be used in fission yeast to visualise interactions between two of the three components of the CMG complex, offering the prospect that this technique could in the future be used to allow studies on replication protein dynamics in living *S. pombe *cells.

## Background

Chromosomal DNA replication in eukaryotic cells requires the complex interplay of a large number of essential and non-essential protein factors in a temporally- and spatially-coordinated manner. Central to the enzymatic machinery at the replication fork is the MCM complex, a heterohexameric DNA helicase composed of the related Mcm2–Mcm7 proteins [1,2]. In yeast, MCM is required for replication initiation at origins and for replication fork progression from origin to non-origin DNA. MCM complexes are loaded onto DNA in inactive form in G1 as part of the pre-replicative complex (pre-RC), become active in S-phase and move with replication forks with the same kinetics as the replicative DNA polymerases [1,2].

How activation of the MCM complex is regulated is not fully understood at present but both post-translational modification (in particular, phosphorylation of individual MCM subunits by the Cdc7-Dbf4 kinase) and interactions with other proteins are believed to have important roles [1]. In the latter context, recent evidence points to the MCM helicase functioning as part of a complex (termed the CMG complex or unwindosome) that comprises the Cdc45 and GINS proteins in addition to MCM [3,4]. The CMG complex possesses helicase activity and itself appears to be a central component of a larger protein structure, termed the replisome progression complex (RPC), that is assembled at replication initiation and disassembled at the end of S-phase [5].

Like MCM, both GINS and Cdc45 are required for the initiation of replication and for replication fork progression [6]. GINS was first identified in functional genomic [7] and genetic screens [8,9] and is a heterotetramer whose four subunits (Sld5, Psf1, Psf2 and Psf3) share a common evolutionary origin and protein fold [10-12]. Single-particle electron microscopy studies [9,13] have suggested that the GINS complex adopts a C-shape structure that is distinct from the compact trapezoid structure determined by crystallographic methods [11,12]. Although the precise molecular functions of GINS and Cdc45 are unknown, it has been shown that GINS is required for formation and maintenance of stable Cdc45-MCM interactions [5] and that recombinant GINS can bind DNA *in vitro *[13].

A detailed understanding of the architecture of the replication fork requires that methods be developed to visualise protein-protein interactions in living cells, ideally in a genetically-tractable organism. Here we apply bimolecular fluorescence complementation (BiFC) to this problem, for the first time using this method in the unicellular fission yeast *S. pombe*. BiFC offers a powerful method for studying protein-protein interactions in living cells [14,15]. BiFC is based on the ability of N- and C-terminal fragments of modified yellow fluorescent protein (YFP) to form a fluorescent complex when brought together by the association of two interacting partners. BiFC provides therefore the opportunity to test for the association of two proteins *in vivo*, and also a method for examining the subcellular localisation of the interacting polypeptides. In contrast to the use of fluorescence energy resonance transfer (FRET) techniques, all this can be accomplished without the need for highly-specialised imaging equipment or sophisticated methods of data analysis [14,15].

Since its initial development and application in mammalian cells [14,16,17], BiFC has been shown to be effective in plants [18,19], in filamentous fungi [20,21] and in budding yeast [22-24]. Here we report the results of the application of BiFC to *S. pombe *for the first time. A series of plasmids has been constructed allowing for convenient C-terminal tagging of fission yeast proteins expressed from their correct chromosomal locations under the control of their native promoters. Genes encoding the Mcm2, Mcm4, Psf1, Psf2 and Cdc45 proteins were then tagged with sequences encoding the N- and C-terminal domains of the engineered YFP protein Venus [25,26]. Using these tagged strains, we demonstrate interaction between Psf1 and Mcm4 proteins in haploid cells and Mcm4 self-association in diploids. Detergent extraction procedures are used to demonstrate that these interactions can also occur on chromatin in binucleate S-phase cells. Our results demonstrate the utility of the BiFC for studying the interactions between replication proteins in yeast cells. A related study reporting the application of BiFC to study interactions between components of the origin recognition complex (ORC) in mammalian cells was recently published [27].

## Results and discussion

**Figure 1:**
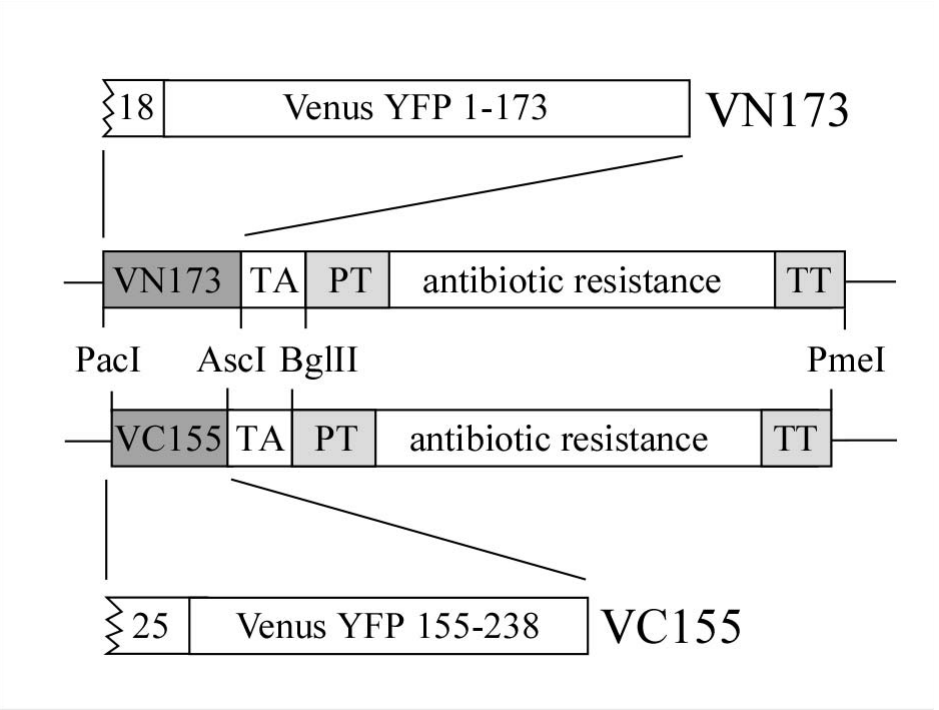
**VN173 and VC155 cassettes for PCR-mediated gene targeting in fission yeast**. Central part: schematic of cassette region of pFA6a-based plasmids showing key features, including restriction sites (*Pac*I, *Asc*I, *Bgl*II, *Pme*I) used for cloning. Abbreviations: TA (*S. cerevisiae ADH1 *transcriptional terminator), PT (*Ashbya gossypii TEF *promoter), TT (*Ashbya gossypii TEF *transcriptional terminator). Upper and lower parts: schematic of VN173 and VC155 Venus YFP fragments. Linker regions are shown (18 and 25 amino acids for VN173 and VC155 respectively).

In order to test whether BiFC could be used in fission yeast, we first constructed pFA6a-based plasmids for PCR-mediated gene targeting in this organism [28]. Open reading frames (ORFs) encoding the N- and C-terminal domains of the engineered YFP protein Venus [25] were amplified by PCR from plasmids pBiFC-VN173 and pBiFC-VC155 [26] and cloned separately into plasmids pFA6a-kanMX6 [28] and pFA6a-natMX6 [29]. The latter carry kanMX6 and natMX6 antibiotic resistance cassettes, conferring resistance in fission yeast to G418 and nourseothricin respectively. Figure 1 shows the schematic structure of proteins expressed from the tagging plasmids.

Using PCR-mediated gene targeting method [28], we attempted to tag genes encoding two of the four subunits of GINS (Psf1 and Psf2), two of the six subunits of MCM (Mcm2 and Mcm4) and Cdc45 in haploid cells. The sequences of the 100^mer ^oligonucleotides used for this purpose are given in Additional file 1. In each case, we attempted to construct both VN173 and VC155 derivatives. Eight of the ten desired strains were obtained by this method without difficulty (see Table 1). Haploids expressing Mcm2-VC155 and Cdc45-VN173 were not obtained, suggesting that the BiFC tag might detrimentally affect protein function in these cases.

**Table 1:** Fission yeast strains

Sp347	h^-S^	[33]
Sp348	h^+S^	[34]
Sp484	*psf1-VN173-kanMX6 *h^+S^	This study
Sp485	*psf1-VC155-natMX6 *h^-S^	This study
Sp486	*psf2-VN173-kanMX6 *h^+S^	This study
Sp487	*psf2-VC155-natMX6 *h^-S^	This study
Sp488	*mcm4-VN173-kanMX6 *h^+S^	This study
Sp489	*mcm4-VC155-natMX6 *h^-S^	This study
Sp490	*mcm2-VN173-kanMX6 *h^+S^	This study
Sp491	*cdc45-VC155-natMX6 *h^-S^	This study
Sp492	*psf1-VN173-kanMX6 mcm4-VC155-natMX6 *h^-S^	This study
Sp493	*psf1-VN173-kanMX6 cdc45-VC155-natMX6 *h^-S^	This study
Sp495	*mcm4-VN173-kanMX6/mcm4-VC155-natMX6 *h^-S^/h^+S^	This study

Next, the tagged strains were mated to one another in an effort to generate the thirteen possible VN173-VC155 combinations for BiFC analysis. The products of the crosses were initially characterised by random spore analysis and later by dissection of meiotic tetrads, with the identity of the strains being determined by testing for sensitivity to G418 and/or nourseothricin and by PCR using specific primers (see Methods). In total, we were only able to isolate double-tagged haploid strains from two of the thirteen possible combinations (summarised in Additional file 2). The viable strains were *psf1-VN173 mcm4-VC155 *and *psf1-VN173 cdc45-VC155*. In all other cases, tetrad dissection showed that the double-tagged strains were inviable (data not shown). The reasons underlying the high degree of synthetic lethality observed in these crosses are unclear. While it is not uncommon for two apparently normal tagged strains to be inviable when crossed together, we cannot rule out the possibility that the lethalities we observe are a direct consequence of the extremely tight binding of the two domains of the YFP protein over long time periods [16] preventing the timely dissociation of the interacting replication factors.

**Figure 2:**
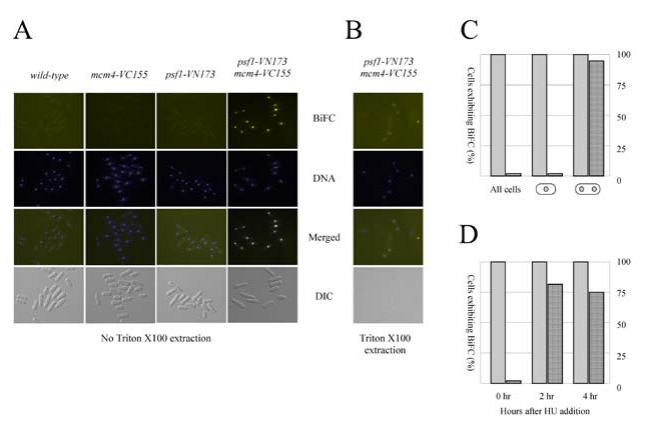
**Visualisation of Psf1-Mcm4 interactions by BiFC**. **A**. Haploid wild-type, *mcm4-VC155*, *psf1-VN173 *and *psf1-VN173 mcm4-VC155 *cells. **B**. Chromatin-associated BiFC signal in detergent-extracted *psf1-VN173 mcm4-VC155 *cells. **C**. Quantitation of nuclear (detergent-extractable) and chromatin-bound (detergent-extraction resistant) BiFC signals in entire population, in uninucleate cells and in binucleate (S-phase) cells. **D**. Quantitation (as above) of nuclear and chromatin-bound BiFC signals in exponentially-growing cells, and in cells treated with 12 mM hydroxyurea for 2 and 4 hours. At least 100 cells were counted for each data point.

To visualise BiFC signals, exponentially-growing cells were examined under the fluorescence microscope (see Methods). No BiFC signal was detected in *psf1-VN173 cdc45-VC155 *cells (data not shown), suggesting that the N- and C-terminal fragments of YFP are insufficiently close to allow YFP complex formation or that topological constraints prevent this from occurring. In contrast, strong BiFC signals could be detected with *psf1-VN173 mcm4-VC155 *but not in either parental strain or in the wild-type (Figure 2A). The fluorescence signal was confined to the nucleus and was present in all cells in the population indicating that the two proteins interact in all phases of the cell cycle and not only during the short period of S-phase. This result was unexpected, as MCM and GINS are not thought to interact with each other outside of S-phase, and may reflect a limitation of the BiFC system, that once the two fragments of YFP are brought together through the interaction of their fusion partners (in this case, Psf1 and Mcm4) their tight binding may, as noted above, prevent subsequent dissociation of the complex, thus preventing investigation of the dynamics of GINS-MCM interactions.

Despite this, we also tested whether we could detect GINS-MCM interactions on chromatin in fission yeast, by treating *psf1-VN173 mcm4-VC155 *cells with detergent to extract non-chromatin-associated material [30], fixing with methanol/acetone and examining them for BiFC (see Methods). Under these conditions, BiFC signals were seen only in binucleate cells (Figure 2B). In fission yeast, S-phase takes place immediately after completion of mitosis in undivided binucleate cells [31]. Approximately 90% of binucleate cells displayed a clear BiFC signal (Figure 2C). Thus the fission yeast Psf1 and Mcm4 proteins are able to interact on chromatin during S-phase.

We also investigated the effect of blocking DNA replication on the Psf1-Mcm4 BiFC signal. To do this, the ribonucleotide reductase inhibitor hydroxyurea (HU) was added to cells in mid-exponential growth and samples analysed for chromatin-associated BiFC following detergent extraction and fixation. By two hours after hydroxurea addition, ~80% of cells displayed chromatin-associated BiFC signals (Figure 2D). Thus, Psf1 and Mcm4 remain associated on chromatin even when replication fork progression is blocked, consistent with results using aphidicolin-stalled replication forks in *Xenopus *egg extracts [4] or in yeast cells [5,32].

To extend these observations, we endeavoured to examine the genetic requirements for the observed BiFC signal by attempting to construct *cut5-T401 psf1-VN173 mcm4-VC155 *and *sld3-10 psf1-VN173 mcm4-VC155 *strains. The temperature-sensitive *cut5-T401 *and *sld3-10 *mutations might both be expected to disrupt the MCM-GINS interaction. However, we found that the *cut5-T401 psf1-VN173 mcm4-VC155 *strain was inviable (confirmed by tetrad dissection) and that *sld3-10 psf1-VN173 mcm4-VC155 *grew extremely poorly, forming microcolonies of very sick cells only after prolonged incubation at 25°C. Growth in liquid YE medium was also extremely poor. As a result it was not possible to examine MCM-GINS BiFC in these cells. We also attempted to construct Mcm4-Psf1 BiFC strains carrying additional mutations in the MCM complex using the cold-sensitive *nda1-KM376 *and *nda4-108 *mutations (*nda1-KM376 *and *nda4-108 *are alleles of *mcm2 *and *mcm5 *respectively). In both cases, the *nda1/nda4 psf1-VN173 mcm4-VC155 *strains were also inviable (K. Saamarthy and S.M., unpublished results). These observations highlight once again the potential pitfalls of using the BiFC system to study the dynamics of essential protein-protein interactions in living cells.

**Figure 3:**
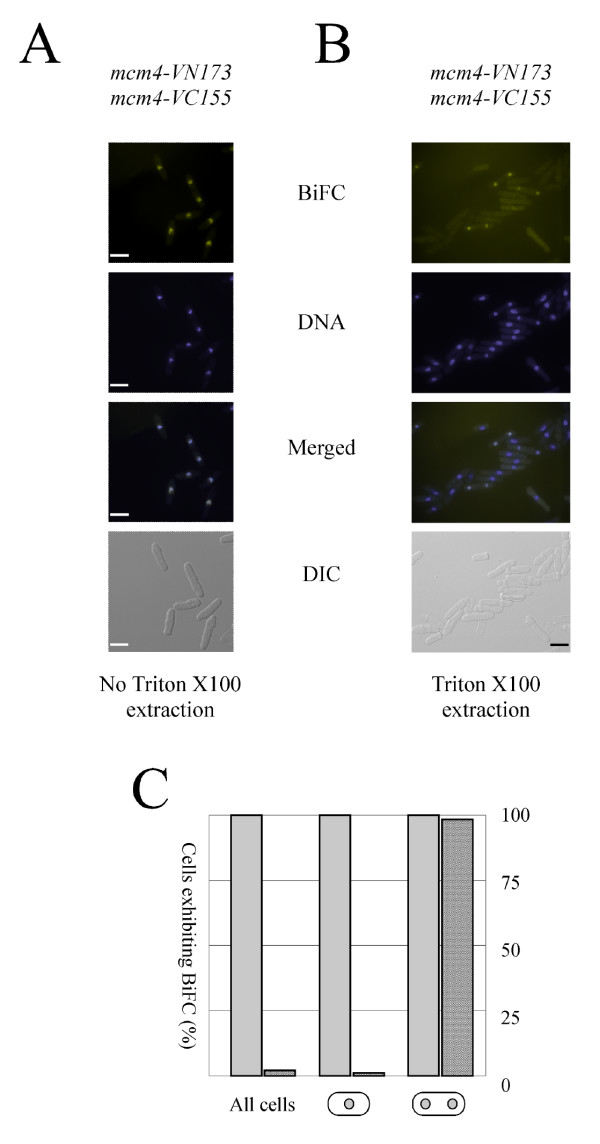
**Visualisation of Mcm4-Mcm4 interactions by BiFC**. **A**. BiFC, DNA and DIC images of exponentially-growing *mcm4-VN173*/*mcm4-VC155 *diploid cells. Bar = 10 μm. **B**. Chromatin-associated BiFC signals in detergent-extracted *mcm4-VN173*/*mcm4-VC155 *cells. Bar = 10 μm. **C**. Quantitation of nuclear and chromatin-bound BiFC signals in *mcm4-VN173*/*mcm4-VC155 *cells. At least 100 cells were counted for each data point.

A number of studies have provided evidence that the MCM helicase is capable of double-hexamer formation and several models for MCM helicase action based on these observations have been presented (reviewed by [1]). The biological significance, if any, of MCM-MCM interactions is unclear, however. To examine whether BiFC could be used as a tool to address this issue, we constructed a *mcm4-VN173/mcm4-VC155 *heterozygous diploid strain by mating *mcm4-VN173 *and *mcm4-VC155 *haploids, neither of which a displayed BiFC signal (Figure 3A and data not shown), and examined these cells under the fluorescence microscope. The *mcm4-VN173/mcm4-VC155 *cells displayed strong nuclear fluorescence (Figure 3A) indicative of Mcm4-Mcm4 interactions *in vivo *in *S. pombe*. Whether this reflects the existence *in vivo *of MCM double-hexamers or merely the close association of MCM single-hexamers within so-called replication factories remains to be determined, however. As with the Psf1-Mcm4 BiFC signal observed in haploid cells (Figure 2B), the Mcm4-Mcm4 signal was also observed on chromatin in binucleate, S-phase cells (Figure 3B, C).

## Conclusion

In summary, we have used BiFC for the first time in fission yeast to demonstrate interactions between the GINS and MCM complexes in the nuclei of exponentially-growing cells and on chromatin in S-phase cells and when replication forks are stalled by nucleotide pool depletion. Interactions between MCM complexes are also seen, with similar characteristics. Whether this system can be usefully exploited to allow analysis of replication protein dynamics in living *S. pombe *cells remains to be seen however, as it is possible that the tight association of the two domains of YFP may interfere with the normal dynamics of the endogenous protein-protein interaction, presenting the risk of experimental artefacts. Significant challenges lie ahead before the system can be validated.

## Methods

### Fission yeast strains and genetic manipulations

The *S. pombe *strains used in this study are listed in Table 1[33,34] with the exception of *sld3-10 *h^-S ^and *cut5-T401 *h^-S ^which were a generous gift of H. Masukata (Osaka University). Standard fission yeast methods were used throughout, except where otherwise indicated. G418 (Invitrogen) and nourseothricin (Werner BioAgents) were used at 100 μg/ml in YE medium. Hydroxyurea (Sigma) was used at 12 mM. Tetrad dissection was performed using a micromanipulator (Singer Instruments). The diploid *mcm4-VN173*/*mcm4-VC155 *strain (Sp495, Table 1) was constructed by mating *mcm4-VN173-kanMX6 *h^+S ^(Sp488) with *mcm4-VC155-natMX5 *h^-S ^(Sp489) for 24 hours at 32°C on malt extract (ME) medium before selecting G418- and nourseothricin-resistant clones on YE plates containing both antibiotics at 100 μg/ml as described above. The presence of both tagged genes in the resulting diploids was confirmed by PCR; the strains were subsequently maintained on YE to prevent sporulation.

### DNA manipulations

Standard molecular cloning methods were used throughout. Enzymes were obtained from New England Biolabs. Oligonucleotides were obtained from DNA Technology A/S (Aarhus, Denmark). 100^mer ^oligonucleotides for PCR-mediated gene targeting were purified by HPLC after synthesis. DNA sequencing was performed commercially by MWG-Biotech AG (Ebersberg, Germany).

### Construction of BiFC plasmids for PCR-mediated gene

Using plasmids pBiFC-VN173 and pBiFC-VC155 as templates [26], the VN173 and VC155 ORFs were amplified by PCR using oligos VN173-5-Pac and VN173-3-Asc (for the VN173 ORF) and VC155-5-Pac and VC155-3-Asc (for VC155 ORF). Oligonucleotide sequences are given in Additional file 1. The PCR products were restricted with *Pac*I and *Asc*I and ligated separately into plasmids pFA6a-GST-kanMX6 [28] and pFA6a-GST-natMX6 [35], from which the *Pac*I-*Asc*I GST region had been removed, to generate the following: pFA6a-VN173-kanMX6, pFA6a-VN173-natMX6, pFA6a-VC155-kanMX6 and pFA6a-VC155-natMX6. The constructs were sequenced to confirm the absence of sequence errors. All four plasmids are available from the authors on request.

### PCR-mediated gene targeting

PCR-mediated gene targeting was performed essentially as described previously [28]. The 100 nt oligonucleotides used for the PCR are listed in Additional file 1. The 3' sequences of the oligonucleotides were identical to those described previously (see oligos for amplification of pFa6a derivatives in Table 1 of Bähler et al., 1998), allowing in-frame fusion of the VN173 or VC155 ORFs at the 3' end of the target genes. Following transformation of either Sp347 (h^-S^) or Sp348 (h^+S^), kanamycin- or nourseothricin-resistant clones were analysed by PCR to identify putative tagged strains. The integrity of the tag was then confirmed by sequencing of the amplified products.

### Fluorescence microscopy

Cells were fixed using methanol/acetone and mounted in 1.2% (w/v) low melting temperature agarose in 10 mM Tris-acetate pH 8.5 at 37°C. For DNA staining, cells were spun down and resuspended in 10 μg/ml Hoechst 33342 (Sigma) for 5 minutes before fixation. Fluorescence microscopy was performed on a Zeiss Axio Imager Z1 microscope (Carl Zeiss, Thornwood, USA). All images were captured with a Plan-Apochromat 100X 1.4 numerical aperture (NA) objective lens. The illumination source was a 100W mercury arc lamp. The following filters were used: for BiFC (41028, Yellow GFP BP) constructs and Hoechst 33342 (31013v2) were from Chroma Technology (Rockingham, USA). Exposure time for BiFC constructs were 9–25 sec. Images were acquired by using cooled Orca-ER CCD camera (Hamamatsu, Japan) and Volocity4 software (Improvision, Lexington, USA) and prepared for publication in Adobe Photoshop (Adobe Systems, Mountain View, USA).

### Chromatin binding assay

Chromatin binding assays were performed as described previously in Kearsey et al. (2005). For analysis of BiFC constructs, an Mg^2+^-containing low salt extraction buffer (20 mM PIPES-KOH pH 6.8, 0.4 M sorbitol, 10 mM potassium acetate, 2 mM magnesium acetate) was used. Cells were extracted at 20°C for 10 min with 10% (w/v) Triton X-100, fixed using methanol/acetone, analysed by fluorescence microscopy as described above.

## Authors' contributions

GA performed almost all the experimental work. SAM devised and directed the project, did a small part of the experimental work, analysed the results and wrote the manuscript. All authors read and approved the final manuscript.

## Supplementary Material

Additional file 1 **Oligonucleotide primers**. Sequences of oligonucleotide primers used in this study. Click here for file

Additional file 2 **BiFC strain construction**. Results of genetic crosses to construct BiFC strains. Click here for file
