# Supplementary material for: MCM-GINS and MCM-MCM interactions in vivo visualised by bimolecular fluorescence complementation in fission yeast
Source: BMC Cell Biol. 2009 Feb 19;10:12. doi: 10.1186/1471-2121-10-12 (PMC2652428; doi:10.1186/1471-2121-10-12)
Supplement: Additional file 1 — Oligonucleotide primers. Sequences of oligonucleotide primers used in this study. [file 1471-2121-10-12-S1.pdf]

Additional file 1: Oligonucleotide primers

A. Primers for plasmid construction

VN173-5-Pac 5′-GTGTGTGGTTTTAATTAAAGTCGACTCTAGAAGATCCATC-3′

VN173-3-Asc 5′-GTGTGTGGTTGGCGCGCCCTACTCGATGTTGTGGCGGATC-3′

VC155-5-Pac 5′-GGTTGTGTGTTTAATTAATACCCGTCCGGCGTGCAAAATC-3′

VC155-3-Asc 5′-GGTTGTGTGTGGCGCGCCTTACTTGTACAGCTCGTCCATG-3′

Restriction sites (*Pac*I or *Asc*I) are underlined.

B. Primers for PCR-mediated gene tagging

MCM2-CTAG-5 5′-TGATTATTGATTCCTTTGTGAATGCCCAAAAAATGAGTGT
TAAACGAAGTTTGTCAAGAACATTTGCTAAATATCTTATTCGGATCCCCGGGTTAATTAA-3′

MCM2-CTAG-3 5′-ATAAAAATGTAAAGCAAATTTTGATTGCAAATTATTAAAG
GATAGATATATGCAACAGATGCAGTAACAAAAGGAGGGTCGAATTCGAGCTCGTTTAAAC-3′

MCM4-CTAG-5 5′-GTGCTTTGGAAAGGCGAGGACGTATTAAGGTTATTACCAG
TGCTGGACATCGCATTGTACGTTCAATTGCACAGACTGATCGGATCCCCGGGTTAATTAA-3′

MCM4-CTAG-3 5′-AAATATGTAAAATATTTTTATAACAAAAAGAACGTGTGTA
TATATAACAAGAAAGACATTCGTATTATGCTCTGTAGTCTGAATTCGAGCTCGTTTAAAC-3′

CDC45-CTAG-5 5′-GTTTTGAAGCAAGTGTGATTGAGTGTCAAAAAAGTGACCT
CGGGGTTTTTTAGAAAGTCTGTCCTTCAAAACACTATTACGGATCCCCGGGTTAATTAA-3′

CDC45-CTAG-3 5′-CTTACATCAGTTGATATCCATACTTATTTACGTGTAATTT
TAATTAGATGAATTAATATTTTCTTTATTAGCAAAATTCCGAATTCGAGCTCGTTTAAAC-3′

PSF1-CTAG-5 5′-ACCTTACTAAAAATTCACAATTGCATGTGCGTGCTACAGA
CGTTGAACGACTCATTGCCCAAGGTTTTTTGGCTAAGTTACGGATCCCCGGGTTAATTAA-3′

PSF1-CTAG-3 5′-ATGGTAGGAATGAATGGCTTATGGATGAAATTTTTAGGGT
CAGTTCAAAAAGCAGTGAAACTCTTATTTGGGAAACAGAGGAATTCGAGCTCGTTTAAAC-3′

PSF2-CTAG-5 5′-TGGAAATTAACGAAATACGTCCTATATTTCGAGAGGTGAT
GGACAGAATGCGCAAAATTGTTCAAGTTTCCCAAGAAGAACGGATCCCCGGGTTAATTAA-3′

PSF2-CTAG-3 5′-ATTTCACTACTACAAAGTTGGTATTCATAAACACTTCGTA
GGATTCATTATCATTATTTTTAAAGTACATCATCCACACGGAATTCGAGCTCGTTTAAAC-3′

Sequences annealing to pFA6a-based plasmids are underlined.

C. Primers for detection and sequencing of tagged genes

MCM2-200	5′-CGATATGAGACGCGAGTCACTGGC-3′
MCM4-200	5′-TGAAGATATGGTCAAAGAACTGGC-3′
CDC45-200	5′-GGTACTTAATAGTTGGAACTTCTA-3′
PSF1-SEQ1	5′-AAGGACGTTGGAGATATTGA-3′
PSF2-SEQ1	5′-ATTAATGAAGTTCAACTAAC-3′
MCM2-150	5′-GCGTCATCTGGAGTCTGCTATCCG-3′
MCM4-150	5′-TATGTTGGTCAGTCAATTGTTAAC-3′
CDC45-100	5′-AGAGGTTGCAAACATGACATCTGC-3′
PSF1-SEQ2	5′-TTGGTCCGAGCTGGATTTGA-3′
PSF2-SEQ2	5′-GGATATCAGGAGGATTTTGC-3′
KANNATMX6-REV	5′-CCCTGCTCGCAGGTCTGCAGCGAGGAGCCG-3′
