# Supplementary material for: MCM-GINS and MCM-MCM interactions in vivo visualised by bimolecular fluorescence complementation in fission yeast
Source: BMC Cell Biol. 2009 Feb 19;10:12. doi: 10.1186/1471-2121-10-12 (PMC2652428; doi:10.1186/1471-2121-10-12)
Supplement: Additional file 2 — BiFC strain construction. Results of genetic crosses to construct BiFC strains. [file 1471-2121-10-12-S2.pdf]

Additional file 2: BiFC strain construction

	psf1-VN173	*psf2-VN173*	*mcm2-VN173*	*mcm4-VN173*
psf1-VC155	-	Synthetic lethal	Synthetic lethal	Synthetic lethal
psf2-VC155	Synthetic lethal	-	Synthetic lethal	Synthetic lethal
mcm4-VC155	Viable (BiFC)	Synthetic lethal	Synthetic lethal	-
cdc45-VC155	Viable (no BiFC)	Synthetic lethal	Synthetic lethal	Synthetic lethal
